# What Does It “Mean”? A Review of Interpreting and Calculating Different Types of Means and Standard Deviations

**DOI:** 10.3390/pharmaceutics9020014

**Published:** 2017-04-13

**Authors:** Marilyn N. Martinez, Mary J. Bartholomew

**Affiliations:** 1 Office of New Animal Drug Evaluation, Center for Veterinary Medicine, US FDA, Rockville, MD 20855, USA; 2 Office of Surveillance and Compliance, Center for Veterinary Medicine, US FDA, Rockville, MD 20855, USA; mary.bartholomew@fda.hhs.gov

**Keywords:** animal pharmacology, pharmacokinetics, data analysis

## Abstract

Typically, investigations are conducted with the goal of generating inferences about a population (humans or animal). Since it is not feasible to evaluate the entire population, the study is conducted using a randomly selected subset of that population. With the goal of using the results generated from that sample to provide inferences about the true population, it is important to consider the properties of the population distribution and how well they are represented by the sample (the subset of values). Consistent with that study objective, it is necessary to identify and use the most appropriate set of summary statistics to describe the study results. Inherent in that choice is the need to identify the specific question being asked and the assumptions associated with the data analysis. The estimate of a “mean” value is an example of a summary statistic that is sometimes reported without adequate consideration as to its implications or the underlying assumptions associated with the data being evaluated. When ignoring these critical considerations, the method of calculating the variance may be inconsistent with the type of mean being reported. Furthermore, there can be confusion about why a single set of values may be represented by summary statistics that differ across published reports. In an effort to remedy some of this confusion, this manuscript describes the basis for selecting among various ways of representing the mean of a sample, their corresponding methods of calculation, and the appropriate methods for estimating their standard deviations.

## 1. Introduction

In setting the foundation for any discussion of data analysis, it is important to recognize that most studies are conducted with the goal of providing inferences about some population. If it were possible to assess every element in a population, inference would be unnecessary. Since it is rarely feasible to evaluate the entire population, measurements are made on a sample of the population and those measurements are used to make inference about the distribution of the variable of interest (e.g., body weight, treatment outcome, drug pharmacokinetics, food intake) in the entire population. Imbedded within the concept of using finite samples to represent the larger population is the requirement that the observations be derived from a randomly sampled subset of the population. The larger the sample size, the better will be the approximation of the population distribution. 

An evaluation of the sample data frequently includes calculation of sample mean and variance. The sample mean is a measure of location. The standard deviation (stdev), i.e., the square root of the variance, provides a measure of the dispersion of the values about the mean [1]. In addition to their use in interpreting individual study outcomes, these values are often compared across studies.

Once a sample mean and variance (or stdev) are calculated, researchers often use these estimates to generate inferences about the population parameters, the true mean (which is the expected value of the population), and the variance. For example, the area under the concentration versus time curve (AUC) is one variable used as an indicator of rate and extent of drug absorption. In a crossover bioequivalence (BE) trial, an AUC is computed for each study subject following the administration of a test product and a reference product and the ratio test AUC/reference AUC is of interest. The natural logarithm of the ratio of test AUC/reference AUC (i.e., the difference in natural log (Ln)-transformed AUC values) is subsequently calculated for each study subject. The mean and residual error (variance) of the within subject test/reference product ratios are determined. On the basis of these statistics, we can generate conclusions as to whether or not two products will be bioequivalent when administered to the population of potential patients. 

While the concept of “mean” may appear straightforward, the “mean” needs to be appreciated from the perspective of the distribution of the underlying data that determined the method by which that mean was estimated. In this regard, depending upon their characteristics (i.e., what they represent), a set of numbers can be described by different mean values. For this reason, it is important to understand the reason for selecting one type of “mean” over another. Similarly, distributional assumptions influence the calculation of the corresponding error (variance) estimates. Data transformations change the distributional assumptions that apply. Nevertheless, there are instances where investigators use transformations when estimating the means but revert back to arithmetic assumptions in the calculation of the variance. Such method for calculating summary statistics is flawed, rending the sample and population inferences derived from that data analysis likewise flawed.

This leads to the question of the consequence of using (or not using) data transformations in the calculation of means and variances. When the data are derived from a normal (symmetric bell curve) distribution, we can infer that about 68% of the population values will be within one stdev of the mean, about 95% of the population values will be within two stdevs of the mean, and 99.7% of the values will be within three stdevs of the mean (the three-sigma rule [1]). We do not have this property to rely upon when using the arithmetic mean and the associated stdev to describe data from any other type of distribution. Appropriately chosen data transformations can result in a distribution that is more nearly normal, that is, closer to a symmetric bell shape, than the distribution of the original data and will permit the use of the three-sigma rule and other benefits that apply to normal data. In other words, when applying the 3-sigma rule, it needs to be applied under the conditions of the transformation that made the data distribution closer to a symmetric bell shaped curve. 

**Figure 1:**
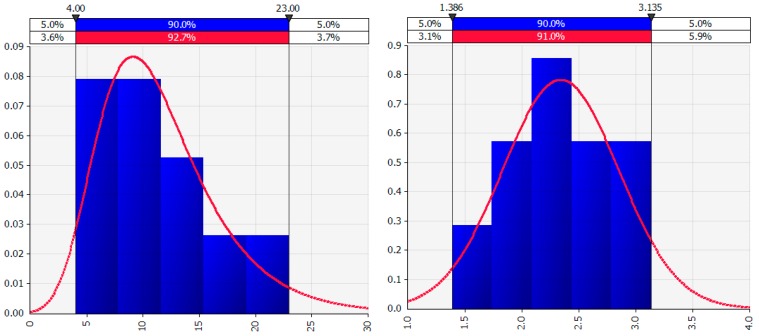
Best fit distribution for “Number” and for “Ln number”.

As an example of how the natural logarithm transformation can correct right-skewed data distributions (a larger proportion of low values) and result in a distribution that is more nearly normal (i.e., closer to a symmetric bell shaped curve), Figure 1 shows the best fit distribution to right-skewed “Number” values in the left graph and the best fit distribution, a normal distribution, to the “Ln number” values in the right graph. The natural logarithm transformation can only be calculated for original values that are non-negative.

Similar results may be shown for the use of the reciprocal transformation (1/”Number”) which applies only to positive values. While there are many data transformations in use, this paper focuses on the natural logarithm transformation and the reciprocal transformation, which are transformations commonly applied to the analysis of right-skewed positive-valued biomedical data.

Taking this example further, we compute the arithmetic mean and the stdevs for both the original “Number” values and the natural logarithm transformed values “Ln number”. The original values are recorded in the units in which they were measured and are familiar to the researcher. However, the “Ln number” values are in units that may be less familiar. As a result, the mean and the mean ± 1, 2 or 3 × stdevs of the Ln-transformed values are transformed back into the original units by exponentiation. When the back-transformed values are compared to those calculated from the original values, we find that the location of the exponentiated mean of the Ln-transformed assessment (i.e., the geometric mean) is shifted to the left of the arithmetic mean. In addition, the distribution of the exponentiated Ln-transformed values is again right-skewed, with larger differences between the mean + stdev than between mean − stdev. While the mean minus some multiple of the stdev may be calculated as a negative number for the untransformed values, the lower limit of the exponentiated Ln-transformed values is constrained to be non-negative. See Table 1. 

Clearly, in the absence of an appropriate data representation (i.e., if and when a transformation of the original observations is appropriate), we will have flawed assessments that will bias our interpretation of the study results and the associated population inferences derived from our analysis. In other words, in the presence of inappropriate data transformations (or in the absence of necessary transformations), the analysis of the data will be biased. It should also be noted that the greater the skewing of the data being evaluated, the greater the magnitude of the difference between the two sets of predictions.

Given the vast differences in inference that might be made depending on the sample statistics used to describe a study’s results, it is evident that authors should specify the nature of the mean being calculated and reported, and that the corresponding stdev needs to be calculated in a manner consistent with the distributional assumptions associated with the calculated mean value.

It is with these points in mind that this educational article provides a review of the different kinds of means, examples of when each may be appropriately applied, and statistically sound methods for estimating the corresponding variances and stdevs. The types of means and their associated stdevs discussed in this article include arithmetic, geometric, harmonic and least squares (see Appendix A for further discussion of these means). We also discuss how to determine the arithmetic mean and variance when data are pooled across published investigations when only summary statistics are available.

Given the density of information covered in this manuscript, we divided the information into the following sections:

2.1. Illustrating the problems: here we provide a summary of the kinds of data or situations where the different methods of estimating the summary statistics may be appropriate. We also provide a table of values to illustrate the differences in the values of the means and stdevs that are estimated (based upon a single set of initial values) based upon the assumption made about the distribution of that data, and we describe how the types of means and stdevs are matched to the data distribution and to the type of question being addressed.

2.2. Within study estimation: ungrouped data: This section applies to the evaluation of means and stdevs for a single set of observations (e.g., the average elimination half-life (*T*_1/2_) for a given drug product). The study design is not intended for a comparison across effects (e.g., no inter-treatment comparison).

2.2.1. Arithmetic mean and stdev.

2.2.2. Harmonic mean and stdev.

2.2.3. Geometric mean and stdev.

2.3. Within study estimation: grouped data: Here we provide an example to showcase the importance of adjusting for unequal number of observations associated with the factors being compared. An example of a method for estimating the mean and stdev in the presence of study imbalance is provided for a simple situation of a parallel study design where the data are assumed to be normally distributed [1,2]. 

2.4. Between-study comparison: There are occasions when reported means and stdevs are combined to obtain a pooled estimate of the mean and stdev of a variable (e.g., AUC). This kind of analysis is frequently used by the generic drug working group within the Clinical and Laboratory Standards Institute (CLSI) Veterinary Antimicrobial Susceptibility Testing Subcommittee (VAST), VET01 document (Performance Standards for Antimicrobial Disk and Dilution Susceptibility Tests for Bacteria Isolated from Animals; Approved Standard) which can be found at the CLSI website, CLSI.org. When generating a cross-study pooling of data, it is important to consider the differences in the number of observations included in each of the pooled study reports. Using a hypothetical example (intentionally different from situations encountered by the CLSI), we provide a method for generating an unbiased estimate of means and stdevs in the presence of study imbalance. Of course, the statistical considerations (as described earlier in this review) need to be evaluated prior to the inclusion of any dataset within a pooled analysis.

It is important to recognize that there are many experimental and statistical considerations to be considered during study protocol development, data analysis, and study interpretation. Among these are estimator bias, independence of observations, distribution of the residuals, and homogeneity of variances. This information is discussed in great detail in basic statistics textbooks [1,2] and therefore will not be covered in this review. 

## 2. Estimation of Means and Variances

### 2.1. Illustrating the Problem

Table 2 provides three types of means (averages) and stdev calculated from one set of values. The issue here is not which is the most appropriate “mean” to use but rather to illustrate that a single set of values can have different “means”, depending upon the assumptions associated with the nature of the distribution from which the data originates. In this example, in addition to use of the untransformed data (i.e., estimation of the arithmetic mean shown in Table 1), the individual values were either transformed to their natural log (Ln) values (resulting in the calculation of a geometric mean shown in Table 1 as the back-transformed value of the mean of “Ln-number”) or as the reciprocals of their original values (resulting in the calculation of a harmonic mean). For example, the value of the first “Number” is 11. When Ln-transformed, the value is 2.4 and when expressed as the reciprocal, the value is 1/11 or 0.09. Within each column, the summary statistics were evaluated based upon the equations provided in this review. The mean estimate, based upon whether the data values in the column are presented on the Ln scale or as a reciprocal and back-transformed from the mean of the values in the column to the original scale, is provided in the corresponding column. That is, the geometric mean is the value obtained, as shown in Equation (11), by exponentiating the arithmetic mean of the Ln-transformed values (2.34, not shown). The harmonic mean is the value obtained by taking the reciprocal of the arithmetic mean of the reciprocal values (0.11, not shown).

The corresponding geometric and harmonic stdevs were calculated in accordance with the methods described later in this manuscript, justified by the use of the Norris expansion method [3]. The percent coefficient of variation (%CV) (where %CV = 100 × stdev/mean), based upon the geometric and harmonic stdevs and corresponding means, is justified by the method we used for calculating the variability estimates. Please note, in contrast with the need to determine the mean ±1, ±2, or ±3 stdev within the scale of the transformation and then report them as their back-transformed values, the %CV is estimated using the means (Equations (1), (3), or (5)) and the formula for the associated stdev (Equations (2), (4), or (12)) described in the following sections (e.g., geometric stdev/geometric mean, harmonic stdev/harmonic mean).

Table 2 showcases the point that an estimate of the mean, stdev and %CV is dependent upon the assumptions made about the characteristics of the data distribution. Accordingly, and in response to confusion that has sometimes been expressed, what appears to be inconsistencies among study reports may in fact be a reflection of how these basic statistical values have been calculated. It is also interesting to note that the arithmetic mean > geometric mean > harmonic mean. A discussion for the mathematical underpinnings for this outcome is provided in Appendix B.

Identifying when each of the kinds of means should be applied is a first-step in this analysis. Indeed, these same points could be important to consider when designing the study as it can influence the number of subjects that need to be included. We leave the issue of study design to the reader, to be matched with the specific objective of the investigation. Within the framework of this review article, a summary of considerations with regard to matching the type of mean with the distribution and study considerations is provided in Table 3.

### 2.2. Within Study Estimation—Ungrouped Data

#### 2.2.1. Arithmetic Mean and Standard Deviation

The arithmetic mean reflects the sum of the individual values divided by the number of values in the sum. In mathematical notation, we show the summing function using a capital sigma (*Σ*) and we indicate that we are summing over all *n* observed values, *x_i_*, by using the index, *i*, to indicate each integer from 1 to *n*, in turn.

(1) $$\bar{X}=\frac{\sum_{i=1}^{n}x_{i}}{n}$$

The stdev is the square root of the sum of the squared differences between each observation and the arithmetic mean, divided by *n* − 1 [1].

(2) $$Stdev=\sqrt{\frac{{\sum_{i=1}^{n}\left(x_{i}-\bar{X}\right)}^{2}}{n-1}}$$

One question that arises is why we divide by *n* – 1 rather than n. Assuming that we have a random selection of observations sampled from the population of interest, dividing by *n* − 1 versus *n*, is necessary to obtain an unbiased estimation of the population variance. This then is because the sample variance is itself a random variable (i.e., if variances were estimated for several sets of samples from the same distribution, we would obtain a range of values). If we were then to take the average of all possible sets of sample variances, the average value should equal the true population variance. Using *n* − 1 is said to account for the fact that one estimate (the sample mean) was used in the calculation of the sample variance. That is also why when one has measurements on the entire population of interest (e.g., the average height of all the students in fifth grade at a particular school in 2016), the population parameter values are obtained for the mean and the variance and the denominator for the variance is no longer *n* − 1 but *n*. In other words, we are no longer calculating sample statistics as population estimates but rather the population values themselves. Since small samples tend to underestimate the variance (stdev), a less biased estimate is achieved by dividing the value by (*n* − 1) rather than *n*.

#### 2.2.2. Harmonic Mean and Standard Deviation

##### Harmonic Mean 

The harmonic mean (HM) is the reciprocal of the arithmetic mean of the reciprocals of the observed values [4]. The reciprocal transformation applies to positive values only.

(3) HM=1(∑i=1n1xin)=n(1x1+1x2+1x3+...+1xn)

Table 3 indicates that the concept of harmonic mean is useful in the evaluation of half-lives. Confusion has occasionally been expressed by the observation that although the arithmetic mean of the individual *T*_1/2_ values is always greater than the HM of the individual *T*_1/2_ values, there is no difference in the estimate of the *T*_1/2_ mean when estimated as 0.693/(arithmetic mean of *λ*_z_) or as the HM of *T*_1/2_ values. The reason for this relationship is detailed in Appendix C.

##### Estimation of the Harmonic Standard Deviation

The formula for the stdev associated with the HM is written as follows [3]:

(4) $${\mathrm{HM}}^{2}\sqrt{\sum_{i=1}^{n}\frac{{\left[\frac{1}{x_{i}}-\frac{\sum_{i=1}^{n}\frac{1}{x_{i}}}{n}\right]}^{2}}{n-1}}$$

where *x_i_* is the original observation and *n* is the number of observations.

Note again that when the stdev for *T*_1/2_ is computed on the basis of the reciprocal *λ*_z_ values, to return to an estimate of the stdev associated with the *T*_1/2_, it is necessary to divide by the constant 0.693. Indeed, it is this estimate of the stdev which should be used when describing the variation of individual values about a HM. The use of stdev values estimated on the basis of the arithmetic *T*_1/2_ is appropriate only when used to describe the variability about an arithmetic mean. Such values should not be reported when the *T*_1/2_ is summarized as the HM. 

The estimation of the stdev associated with the HM, and the corresponding Excel codes, are provided in Table 4.

#### 2.2.3. Geometric Means and Standard Deviations

##### Estimation of the Geometric Mean

The geometric means are typically reported when describing data that have been Ln-transformed prior to analysis (e.g., AUC or *C*_max_). It is also used for the estimation of means when the values are multiplied rather than added together. Both of these situations are described below.

##### Multiplicative Relationships

The geometric mean is calculated as the *n*^th^ root of the product (denoted by the symbol Π) of the *n* positive observations.

(5) $$G=\sqrt[{n}]{\Pi_{i=1}^{n}x_{i}}={\left(x_{1}x_{2}\cdot \cdot \cdot x_{n}\right)}^{1/n}$$

To illustrate the error that would have occurred if the arithmetic (additive) rather than the geometric (multiplicative) mean was estimated when dealing with multiplicative relationships, we can consider the growth rate values provided in Table 5 (includes Excel code). Here we see that by calculating the arithmetic rather than the geometric mean, we would have markedly over-estimated the proportion of initial amount remaining (89.2% versus the 76.9%). Examples where this kind of calculation may be applicable include bacterial growth (where we are dealing with a constant time for change measurement) or in a financial situation where we are concerned with changes as a function of month or year.

A slightly different set of considerations would be needed if the time associated with the rates of change is not held constant. For example, consider the situation where geometric bacterial growth rate is being traced in response to changing ecological conditions. The numbers of bacteria after a period of change can be described as follows:

*Y* = *X* × *Δ*^*τ*/*λ*^ (6)

where *X* = the starting bacterial number

*Δ* = the kind of change (where Δ = 2 for doubling; Δ = 0.5 for halving)
*λ* = the time associated with a doubling or halving of bacterial number
*τ* = the duration of time
*Y* = the resulting bacterial number

In this hypothetical example, we are starting with 100 isolates. These bacteria have an initial doubling time of 10 min for a duration of 20 min, a reduction in numbers (e.g., due to the presence of some stressors) where there is a halving of numbers every 35 min for a duration of 120 min, followed by a resumption of growth at the original growth rate for 60 min. Sequentially using the equation above, the estimated number of bacteria at the end of 200 min is found to be 2377.6:

*Y*1 = 100 × 2^20/10^ = 400 (7)

*Y*2 = 400 × 0.5^120/35^ = 37.15 (8)

*Y*3 = 37.15 × 2^60/10^ = 2377.6 (9)

The results are also depicted in Table 6 (values) and Table 7 (Excel spreadsheet code):

What was the average rate of change that occurred over this 200 min. duration? Can the formula for computing the geometric mean be applied to these three relative changes to obtain a meaningful geometric mean? 

The answer to that is NO, and here is why. As expressed in this example, the three relative changes are not comparable expressions of rate of change. For the computation of the geometric mean to be meaningful, the relative changes must all be expressed for the same time interval.

In the financial example, the rate of increase was given in annual increments. In the bacterial growth example, the doubling time was expressed as a 10 min interval while the halving time is expressed as a 35-min interval. Thus, one of the rates needs to be converted to the time interval of the other. As an illustration, the rate of decrease during a 10-min time interval is determined by taking τ to be 10. Therefore, the relative decrease in a 10-min interval is:

Δ^*τ*/*λ*^ = (0.5)^10/35^ = 0.820 (10)

Following the format of the financial example above, there are two 10-min intervals with a relative change of 2, twelve 10 min intervals with a relative change of 0.820, followed by six more 10-min intervals with a relative change of 2. The geometric mean of the relative change per 10-min interval is (2^2^ × 0.820^12^ × 2^6^)^1/20^ = 1.172. Table 8 shows the calculation of the expected number of bacteria at the end of each 10-min interval and the calculation of the geometric mean of the 10-min interval rates. In addition, the table shows that replacing each 10-min interval rate with the geometric mean of the 10-min interval rates results in the same calculation of number of bacteria at the end of 200 min.

**Figure 2:**
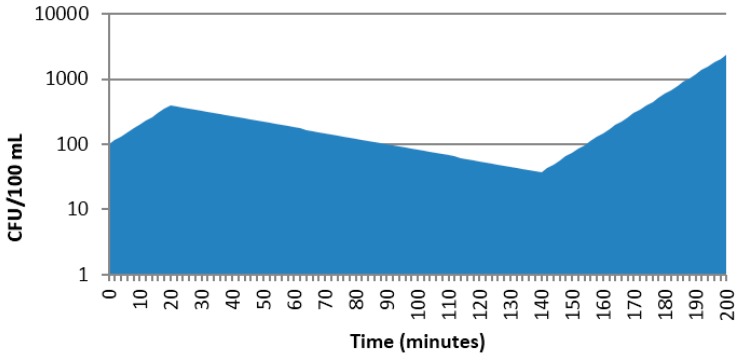
AUC for estimating average CFD over time.

Note that if one wanted to estimate the mean number of colony forming units (CFUs) over the 200 min duration (i.e., mean CFU/min), this would be estimated by integrating the CFU vs. time profile (area under the curve, AUC), with the total area divided by 200 min (Figure 2). Concepts of AUC are familiar to scientists working with pharmacokinetic data. Since the AUC_0–200_ ~ 57982, the mean CFU/min ~ 290.

##### Ln-Transformed Data

This discussion of geometric means from the example of rates, as shown in the bacterial example, segues with the estimation of means when using Ln-transformed parameters such as AUC and *C*_max_. Ln-transformation of these pharmacokinetic parameter values is based upon an assumption that they are better described in terms of a log-normal rather than a normal distribution. Because Ln(a) + Ln(b) = Ln(ab), the process of adding Ln-transformed values is in fact a multiplicative computation. Furthermore, because Ln (ab)^1/*n*^ = (^1^/*n*)Ln(ab) and exp[Ln (ab)^1/*n*^] = (ab)^1/*n*^, exponentiation of the averaged Ln-transformed values reverses the estimation procedure from one of addition (Ln-transformed values) to that of multiplication when expressed on the original scale.

In mathematical symbols, the geometric mean from Equation (5) may be obtained by:

(11) exp[∑i=1nln(xi)n]=(x1x2⋅⋅⋅xn)1/n

In the situation where the original data are log-normal and log transformed, it is convenient and appropriate to obtain a value for the geometric means by exponentiating the arithmetic average of the log-transformed values.

Using the values provided in Table 2, let us this time assume that rather than *T*_1/2_, these reflect *C*_max_ values (Table 9). The difference between the arithmetic mean of the untransformed data versus the geometric mean (based upon the Ln-transformed dataset), is provided below. Again, the arithmetic mean of the Ln-transformed data are exponentiated to obtain the geometric mean (i.e., 10.4 = exp^2.34^).

##### Estimation of the geometric standard deviation (for Ln-transformed values)

The following equation describes a method for obtaining an estimate [3] of the stdev when the geometric mean, *G*, is used:

(12) $$G\times \sqrt{\frac{\sum_{i=1}^{n}{\left[\ln(x_{i})-\frac{\sum \ln(x_{i})}{n}\right]}^{2}}{n-1}}$$

where the *x_i_* are the individual observations, with *i* ranging from 1 to *n* [4].

The calculation of the stdev for the *C*_max_ example from Table 9 is provided in tabular form (values and Excel equations) in Table 10.

### 2.3. Least Square (Marginal) Means Grouped Data

This discussion focuses on the estimation of means and stdevs when there are an unequal number of observations associated with the factors being compared [5,6]. While the precise calculation of the marginal means need to reflect the study design and the statistical model being used, we provide a simple case of two sets of independent observations associated with the effects being compared. We are also assuming that the data are normally distributed for the purpose of this example. 

#### 2.3.1. Estimation of the Mean 

There are circumstances when the study design necessitates blocking the experimental units by a specified variable (e.g., gender, age, sequence of treatment administrations) due to expected differences among those experimental units that are attributable to the specified variable [6]. Under the conditions where there are an equal number of observations within each block, sample statistics may be based upon arithmetic means and variances. However, a more complicated situation is when there is study imbalance such that some blocks of experimental units are under-represented relative to other blocks, e.g., more males than females.

Least square means (LSmeans, also known as estimated population marginal means) provide an opportunity to obtain an unbiased estimate of averages in the face of this kind of study imbalance. For example, Table 11 provides a hypothetical example of body weights measured in two groups of men and women. In one group, all observations were made in the individuals that were enrolled in a daily exercise routine. The other group was comprised of individuals who did not engage in any regular exercise program. Due to differences in the proportion of men and women enrolled in these two groups (a parallel study), males had the greater total influence on results of the arithmetic mean generated in the exercise group (more males than females) while females had the greater influence on the arithmetic mean value calculated in the no exercise group (more females than males). For this reason, when looking at the arithmetic means (with and without exercise), the results suggest that body weights were actually lower in the no exercise group as compared to that associated with people who exercised. However, when averaging the means within each cell (i.e., the marginal means determined, for example, by taking the average of males in the exercise group and the average of the females in the exercise group) and then basing our LSmean estimates for the exercise group and the no exercise group on the average of the corresponding marginal means, we now see that on the average, body weight was slightly lower in those individuals that exercised than those that did not. That is, let ${\bar{X}}_{1}$ be the marginal mean for the males in the no exercise group, let ${\bar{X}}_{2}$ be the marginal mean for the females in the no exercise group, and similarly, let ${\bar{X}}_{3}$ be the marginal mean for the males in the exercise group, and ${\bar{X}}_{4}$ be the marginal mean for the females in the exercise group. Then, the LSmean for the no exercise group, T0 is:

(13) $${\bar{X}}_{T0}=\frac{{\bar{X}}_{1}+{\bar{X}}_{2}}{2}$$

#### 2.3.2. Estimation of the Stdev about the LSmean 

Inherent in the use of LSmeans [6,7] is an assumption that there is some difference between the groups comprising the two treatments being compared (in our example, the difference between the groups is reflected by the separation of participants by gender). Under that assumption, we need to consider if the mean and stdevs reflect a normal distribution or some other type of distribution. With respect to the example above, we see that men and women clearly have unequal body weights, irrespective of whether or not they were on the exercise program. Thus, the means and, possibly, the variances will likewise be dissimilar. It is for this reason that the LSmeans for the treatment effects, which in our example corrected for the imbalance of gender blocks within treatment, provides a different result than did the arithmetic treatment mean. 

For this reason, estimating the variability about the LSmean is far more complex than those associated with harmonic, geometric, or arithmetic means. This is because the estimation of variability for the LSmean needs to consider both the variability within a block and between blocks. Therefore, these are typically generated through using an analysis of variance (ANOVA) program. When running an ANOVA, the value that is reported with the LSmeans is the standard error (SE). Therefore, the question is “How does a statistical program estimate the LSmeans and the corresponding error about the mean estimate?” To begin, let us consider the equation for calculating the SE of an arithmetic mean. When dealing with a simple random sample taken from the population of interest, the SE of the mean is the square root of the variance divided by the sample size (which is equal to the stdev divided by the square root of the sample size).

(14) $$SE\;\mathrm{of}\;\mathrm{the}\;\mathrm{mean}=\sqrt{\frac{\mathrm{variance}}{n}}=\frac{\mathrm{Stdev}}{\sqrt{n}}$$

In this case, if one is given the *SE* of the mean, simple multiplication of that value by the square root of the sample size yields the stdev estimate for the underlying population of values. In other words, if we repeatedly sampled and obtained means derived from the same population, the deviation of estimates of the average of those means (i.e., the stdev about the mean of the means) can be described by the *SE* of the mean, and the variability among the estimates of the means will be less than the variability associated with the individual observations.

**Figure 3:**
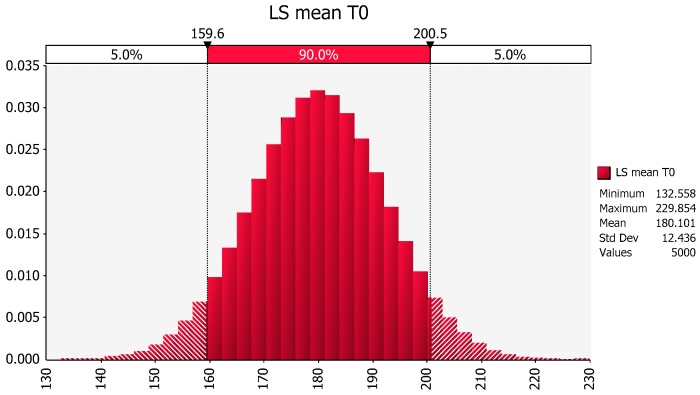
The distribution of weight values for the no exercise group predicted using the LSmean and a stdev estimate based on the standard error (SE) of the LSmean when assuming that the sample was generated from a single normal population.

For example, the LSmean for the no exercise treatment (T0) values is the average of the marginal mean of the 10 females (158 lbs) and the marginal mean of the 5 males (202 lbs). This results in the LSmean of 180 lbs, with a computer-generated SE for this LSmean being 3.21 lbs. Upon multiplying 3.21 lbs by the square root of 15 (the total number of observations in the no exercise group (5 males + 10 females), we estimate the stdev of the no exercise group to be 12.43 lbs. Based on this information, we would imagine the underlying population that generated this data summary to look as follows (Figure 3).

However, given that we know that there are differences between men and women, we need to ask whether such a variability estimate provides an accurate representation of the true population. Before reporting these error estimates, we need to ask how to provide a close approximation of the underlying population(s) from which we obtained our samples. In fact, it is with that question in mind that an ANOVA was performed with a model that accounted for both gender and treatment effects. 

**Figure 4:**
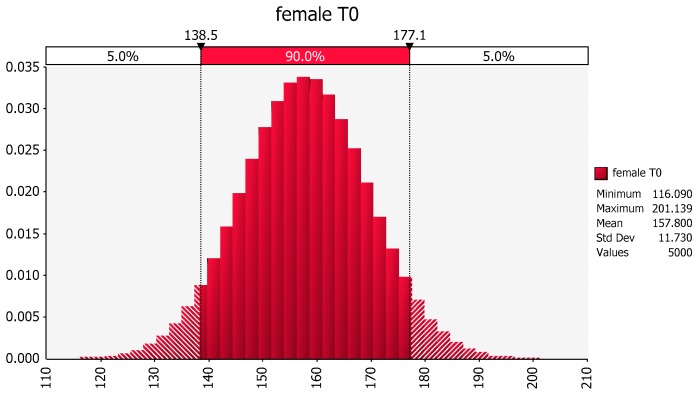
Distribution of female weights based upon the mean and variance of the sample values of body weights of females in the no exercise group. The *X*-axis represents body weight (lbs) and the *Y*-axis represents the fraction of the population of females at that body weight based upon the information contained within subset of individuals included in this hypothetical study. The possible values of the *Y*-axis range from a value of zero (no individuals expected at that body weight) to 1 (all individuals in the population will have the identical body weight).

Revisiting the same dataset, we see that the distribution of weights in females that did not exercise is about normal (157.8, 11.73), as shown in Figure 4.

**Figure 5:**
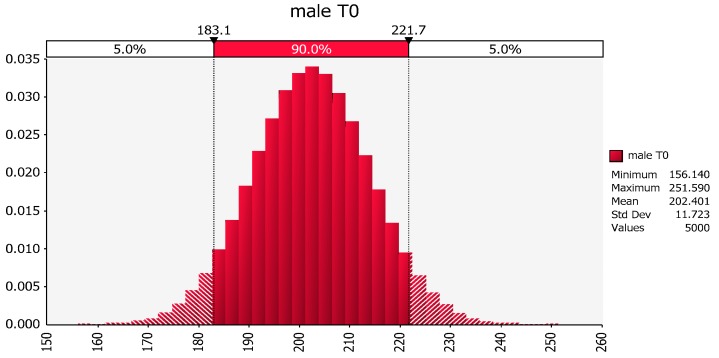
Distribution of male weights based upon the mean and variance of the sample values of body weights of the males in the no exercise group.

Similarly, the distribution of weights in the males that did not exercise is about normal (202.4, 11.72), as shown in Figure 5.

**Figure 6:**
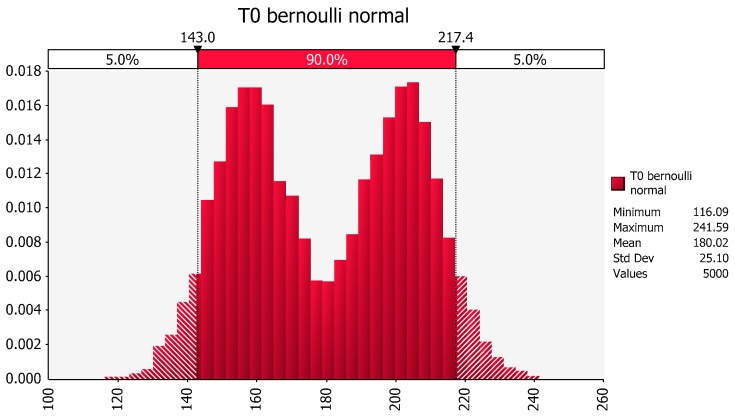
Distribution of population of male and female body weights based upon the mean and variance of the sample values from the no-exercise group, assuming an equal likelihood of sampling from either gender.

In requesting an estimate of the least squares mean for treatment in the no exercise group, we are essentially requesting an estimate of the mean that would have been obtained if we had sampled from the male population of non-exercisers and from the female population of non-exercisers equally. Figure 6 provides the distribution that results when we have an equal probability of drawing a value from the male distribution and from the female distribution. Note that the population is no longer represented as a single normal distribution but rather as a bimodal equal mixture of the male and the female distributions. 

As evidenced by this figure, and as reflected in the corresponding estimation of the stdev, this bimodal distribution has a higher estimate of the stdev of T0 (25.10) as compared to that reflected by the previous assumption that the mean was associated with a single normally distributed population (stdev of T0 = 12.44). This underscores the importance of considering the underlying assumption that is the foundation for the data analysis. Within the context of this example, having generated a statistical analysis using a model containing a term for gender effects and having discovered that gender is a statistically significant term, one needs to determine the information that should be conveyed about the LSmeans when combining the two genders and the stdev for the underlying joint population. In light of the identified gender effects, the question is whether it was more appropriate to compare treatments (with or without exercise) using data that are pooled across sexes or whether it would have been preferable to separate the assessment to one generated within a gender. The answer to that question depends upon the study objective which in turn, should be reflected in the study protocol.

How does this relate to our typical pharmacokinetic or bioequivalence studies? This kind of question may have relevance when there is reason to believe that there may be gender-by-formulation interactions. Unless there are data to the contrary, however, we typically assume that the two subpopulations do not differ with respect to their overall mean values and variances. Another example of potential relevance is imbedded within the design of crossover trials where there is the assumption of no statistically significant sequence effects (i.e., that the relationship between the rate and extent of absorption for two treatments do not differ as a result of the order in which they are administered). Accordingly, even in the face of an imbalance in subjects nested within sequence, we allow for the use of the ratio of the LSmeans and the corresponding SEs of that estimate when calculating the 90% confidence intervals for AUC and *C*_max_. Nevertheless, this exercise underscores why, from a statistical perspective, calculation of the 90% confidence interval could be biased in the face of statistically significant sequence effects.

For each main effect LSmean, Table 12 provides a comparison of the corresponding stdev estimated by using the within/between calculation vs. that estimated by simulating the population as if there were an equal probability of drawing from the two groups comprising the LSmeans. On the outside chance that the stdev for the bimodal situation is desired, it can be approximated by using the within/between type of calculation used for the meta-analysis example described in the next section (estimating the variance when utilizing data derived across several studies).

### 2.4. Cross Study Comparisons

Research based on synthesizing information from a number of independently conducted biomedical studies has been occurring since at least 1904 when Karl Pearson compared infection rates among inoculated soldiers and soldiers that had not been inoculated [8]. In the 1930s, as related in the Hodges and Olkin [9], publications on how to combine data from agricultural experiments were written by groundbreakers in the field of statistics, L.H.C. Tippett, R.A. Fisher, Karl Pearson, Yates and Cochran. From there, the synthesis method (that came to be known as the meta-analysis) expanded into the social sciences and has experienced a burgeoning popularity in the areas of clinical medicine and epidemiology. Accompanying the increase in its application, there was a concomitant growth in the numbers of papers that provided complex methodologies for combining information from various studies.

When pooling information across studies to estimate an overall average and variance, there needs to be consideration given to the number of observations in each of the respective studies, the within-study variance associated with each investigation, and the cross-investigational variance. In situations where this numerical solution would be employed, we may have access only to published sample statistics such as the sample size, mean and stdev from each of studies evaluated. We may not have access to the underlying datasets. Therefore, there is a need to ascertain an appropriate method by which to estimate means and variances when using conducting a cross study evaluation based solely on the available summary statistics.

To address this question, let us consider a scenario where there were four trainers, each one having a different number of runners under their tutelage. A separate study was conducted for each of these trainers to ascertain the average miles per hour (MPH) and the stdev among runners covering a given course. Their corresponding metrics are summarized below (Table 13):

If the goal is to estimate a global stdev and mean (i.e., to describe the MPH for all runners, irrespective of their trainer), the data generated from each of these studies would need to be combined. The synthesis of the information derived from each of the four trainers is contingent upon an assumption that the samples were drawn from the same underlying population (i.e., runners for the four trainers were derived from the identical population of potential runners). In other words, we assume that the differences in the statistics among the studies reflect only sampling variation. It is only on the basis of that assumption, that the cross study mean and stdev can be calculated as described below.

#### 2.4.1. Estimating the Population Mean

The index for study number (the trainer in this example) is *i*, going from 1 to *I*, the number of studies. *I* is four in this example. For study *i*, we have the sample size *n_i_* (i.e., the number of subjects included in study *i*), a sample average ${\bar{X}}_{i}$, and a standard deviation *stdev_i_*.

In many cases, published manuscripts provide a biased estimate of cross study mean and stdev because differences in samples sizes are not considered. It is not uncommon to find the study averages, or cross-study mean, used to estimate the population mean. In so doing, each observation is not receiving an equal weight (influence) on the total population average. Accordingly, as shown in Equation (15), the mean is the unequally weighted (uneqwtd) mean.

(15) $${\bar{X}}_{..uneqwtd}=\frac{\sum_{i=1}^{I}{\bar{X}}_{i}}{I}$$

In this example, it is important to recognize that what is being described is the average of the individual study (trainer) averages and not the averages across all of the individual observations. When the population mean estimate is calculated in this way, individual observations from different studies exert an unequal influence on the overall estimate. For example, if one study contains data on 4 subjects while another study includes 20 subjects, then the simple averaging of the two studies would result in each subject in Study 1 contributing ^1^/_8_^th^ (^1^/_4_ × ^1^/_2_) of the overall influence on means and variances while each subject in Study 2 would contribute only ^1^/_40_^th^ (^1^/_20_ × ^1^/_2_) of the overall influence. We need to adjust the values so that each subject in each study contributes only ^1^/_24_^th^ to the estimate of the population mean.

To weight each subject equally in the population mean estimate, the LSmean should be used.

In this example, *N* = 35 (10 + 5 + 8 + 12 runners).

(16) $$\bar{X}..=\frac{\sum_{i=1}^{I}n_{i}{\bar{X}}_{i}}{N}\;\mathrm{where}\;N=\sum_{i=1}^{I}n_{i}$$

So in our trainer example, the overall LSmean would equal:

[(10 **×** 6.2) + (5 **×** 5.5) + (8 **×** 6.1) + (12 **×** 6.8)]/35 (17)

The calculations are provided in the two right hand columns of Table 13. 

#### 2.4.2. Estimating the Population Variance

Recognizing that the stdev is the square root of the variance, we will first consider estimating the global stdev since the stdevs are given for each study. 

Each standard deviation, *SD_i_* estimates the (square root of) variation within the study from which it was calculated. Just as with the samples averages, one might think of estimating the population within study stdev as the average of the sample stdevs, deriving an average within-study standard deviation (*SD_WA_*) as follows.

(18) $$SD_{WA}=\frac{\sum_{i=1}^{I}SD_{i}}{I}$$

Similarly, one might think to estimate between-study standard deviation (*SD_BA_*) as the stdev among the study means as follows:

(19) $$SD_{BA}=\sqrt{\frac{{\sum_{i=1}^{I}\left(\bar{X}..-{\bar{X}}_{i}\right)}^{2}}{I-1}}$$

The concept of partitioning total variance into both within study and between study variations is familiar to anyone who has studied the analysis of variance. Clearly, using the square of just one of *SD_WA_* or *SD_BA_* will seriously underestimate the total population variance. The most straightforward way to think about calculating an estimate of the population variance from the estimates of within and between study stdevs would be to square the stdevs and to add them.

(20) $$TotalVar_{unwtd}={\left(SD_{BA}\right)}^{2}+{\left(SD_{WA}\right)}^{2}$$

Doing so, however, results in a sum which, much like the unweighted mean in Equation 15, does not appropriately weight the contribution of the within and between study components of variance to the total, nor the magnitude of that contribution for each study within the two components. Therefore, to calculate the estimate of population variance with the correct weights, we follow the steps as described in Appendix D. Accordingly, the within study sums of squares, *SS_w_* is the weighted sum of the individual study variance estimates.

(21) $$SS_{W}=\;\sum_{i=1}^{I}\left(n_{i}-1\right)\;SD_{i}^{2}$$

The between-study sums of squares, *SS_B_*, is the weighted sum of the squared deviations of the individual study means from the LSmean.

(22) $$SS_{B}\;=\sum_{i=1}^{I}n_{i}\left({\bar{X}}_{i}-{\bar{X}}_{..}\right){\;}^{2}\;$$

Then the estimate of the population variance is the total sums of squares divided by the total sample size minus one.

(23) $$Variance_{WB}\;=\frac{\left[SS_{W}+{SS}_{B}\right]}{N-1}=\frac{SS_{T}}{N-1}$$

The weighted total stdev is the square root of the weighted variance estimate (Table 14) and the corresponding Excel codes are provided in Table 15. A discussion of the numerical underpinnings of this approach is provided in Appendix D.

## 3. Closing Comments

Our hope is that this educational article will serve both as a resource and as an instructional tool for the scientific community. Where possible, we provide samples of excel coding so that an estimation of the respective means and stdevs can be readily transferred to an Excel spreadsheet. 

Importantly, through the discussions in this manuscript, our goal is to encourage an appreciation and identification of the underlying assumptions that influence our interpretation of any dataset.

## Figures and Tables

**Table 1:** Comparison of statistics based on original values and Ln-transformed values back-transformed to the original units. Stdev: standard deviation.

Transformation Used	Mean	Mean ± Stdev	Mean ± 2 Stdev	Mean ± 3 Stdev
Untransformed	11.6	5.7, 17.3	0.2, 23	−5.5, 28.7
Back-transformed from Ln	10.4	6.2, 17.3	3.7, 28.8	2.2, 47.9

**Table 2:** Example of differing summary values depending upon the underlying assumptions and estimation procedures for obtaining the mean.

	**Number**	**Ln number**	**1/Number**
	11	2.4	0.09
	7	1.95	0.14
	9	2.20	0.11
	4	1.39	0.25
	10	2.30	0.10
	12	2.48	0.08
	23	3.14	0.04
	15	2.71	0.07
	7	1.95	0.14
	18	2.89	0.06
**Type of estimate**	**Arithmetic**	**Geometric**	**Harmonic**
Mean	11.60	10.38	9.20
Stdev	5.70	5.29	5.06
%CV	49.14	50.96	50.03

**Table 3:** Matching the selection of mean to the nature of the distribution and the question being addressed.

Type of mean and standard deviation	Nature of distribution (examples)	Considerations for its application
Arithmetic	Normal (i.e., additive)	When the estimate of interest is based upon the sum of the individual observations and the data are best presented as a normal distribution.
Harmonic	Reciprocal transformation of positive real values	In general, the harmonic mean (HM) is useful for expressing average rates (e.g., miles per hour; widgets per day). In clinical pharmacology, an elimination rate constant, *λ*_z_, is estimated for each subject based on his/her systemic drug concentration during the depletion portion of the Ln-concentration vs. time profile. The corresponding estimate of the elimination half-life (*T*_1/2_) is then derived on the basis of the elimination rate constant (see Appendix C for more details on calculating the terminal elimination rate where we show that *T*_1/2_ = Ln 2/*λ*_z_ or 0.693/*λ*_z_). Because of the relationship of *T*_1/2_ to the reciprocal value of *λ*_z_ (the latter being a rate constant), harmonic means may be used when describing the average time to reduce the systemic drug concentrations by 1/2. It should be noted that if the mean *T*_1/2_ was generated by obtaining the arithmetic average of the *T*_1/2_ values, then that estimate should be referred to as an arithmetic mean and not as a HM. The mean *T*_1/2_ represents a HM only when the actual averaging was done on the basis of *λ*_z_ estimates. It is only in that case (i.e., when the mean *T*_1/2_ was derived via transformation of the HM of *λ*_z_) that we have the HM of *T*_1/2_.
Geometric	Log transformation of positive real values	Within the realm of pharmacokinetics, geometric means are typically used when describing the means of variables such as area under the curve (AUC) and maximum concentrations (*C*_max_). These variables are often transformed to the natural logarithm (Ln) prior to analysis and the geometric mean computed using the back-transformation shown in Equation (11). This type of assessment is also important for estimating the average performance of an investment where the interest rates are compounded over time or when averaging changes in bacterial growth rates.
Least square means	Should be consistent with the distribution characteristics of the data collected and the model used to address the study assumptions and investigation	The use of least square means is important when there are an unequal number of observations associated with any of the terms in the statistical model.

**Table 4:** Comparison of arithmetic vs. harmonic standard deviation (Stdev) values and Excel codes for *T*_1/2_.

	Data Row	Column C	Column D	Column E	Column F
		*T*_1/2_	*λ*_z_	*T*_1/2_ sq′d Deviations	λ_z_ sq′d Deviations
	Row 2	11	0.063	(C2–C12)^2^	(D2–D12)^2^
	Row 3	7	0.099	(C3–C12)^2^	(D3–D12)^2^
	Row 4	9	0.077	(C4–C12)^2^	(D4–D12)^2^
	Row 5	4	0.17325	(C5–C12)^2^	(D5–D12)^2^
	Row 6	10	0.0693	(C6–C12)^2^	(D6–D12)^2^
	Row 7	12	0.05775	(C7–C12)^2^	(D7–D12)^2^
	Row 8	23	0.05775	(C8–C12)^2^	(D8–D12)^2^
	Row 9	15	0.0462	(C9–C12)^2^	(D9–D12)^2^
	Row 10	7	0.099	(C10–C12)^2^	(D10–D12)^2^
	Row 11	18	0.077	(C11–C12)^2^	(D11–D12)^2^
Row 12	Arithmetic mean	11.6	0.0753		
Row 13	Harmonic mean of *T*_1/2_		9.20		
	Arithmetic Stdev	5.70		sqrt[(sum(E2:E11))/9]	
	Harmonic Stdev		5.06		[D13^2^ × sqrt[(sum(F2:F11))/9]]/0.693

**Table 5:** Comparison of estimating average change when expressed as an arithmetic versus as a geometric mean when there are compounded (multiplicative) changes.

	**Data Row**	**Interest (percent)**	**Column D relative change**	**Excel code for geometric mean**
	Row 2	0.25	1.25	-
	Row 3	−0.36	0.64	
	Row 4	0.18	1.18	
	Row 5	0.14	1.14	
	Row 6	−0.75	0.25	
Arithmetic mean	-	−0.108	-	
Geometric mean			0.769	(D2 × D3 × D4 × D5 × D6)^(1/5)^
Proportion remaining	-	0.892	-	-

**Table 6:** Estimation of bacterial growth.

Doubling time or halving time (*λ*)	Time duration (*τ*)	Value of “*X*”	Relative change (*Δ*^*τ*/*λ*^)	Resulting # bacteria (*Y*)
10	20	100	4	400
35	120	400	0.0929	37.150
10	60	37.15	64	2377.591

**Table 7:** Excel spreadsheet code for estimating bacterial growth rate.

Data Row	Column A	Column B	Column C	Column D	Column E
	Doubling time or halving time	Time duration	Value of “*X*”	Relative change	Resulting # bacteria (*Y*)
Row 1	10	20	100	2^B1/A1^	C1 × D1
Row 2	35	120	400	0.5^B2/A2^	C2 × D2
Row 3	10	60	37.15	2^B3/A3^	C3 × D3

**Table 8:** Calculating the number of bacteria at 10-min intervals. GM: geometric mean.

Column A	Column B	Column C	Column D	Column E	Column F	Column G
Row	Minutes	10 min change rate change (*∆*)	Number of bacteria (*Y*) based on 10-min change	Estimating *Y* [*X* × *∆*^*τ*/*λ*^ where *τ* = 10 and *λ* = 10]	Number of bacteria based on geometric mean	*X* × GM (*τ*/*λ*)
2	0		100			
3	10	2	200	D2 × C3	117.17	D2 × $C$26
4	20	2	400	D3 × C4	137.28	F3 × $C$26
5	30	0.82	328.13	D4 × C5	160.85	F4 × $C$26
6	40	0.82	269.18	D5 × C6	188.46	F5 × $C$26
7	50	0.82	220.82	D6 × C7	220.82	F6 × $C$26
8	55		200.00	D7 × (0.82^0.5^)		
9	60	0.82	181.14	D8 × (C9^0.5^)	258.73	F7 × $C$26
10	70	0.82	148.60	D9 × C10	303.14	F9 × $C$26
11	80	0.82	121.90	D10 × C11	355.18	F10 × $C$26
12	90	0.82	100.00	D11 × C12	416.16	F11 × $C$26
13	100	0.82	82.03	D12 × C13	487.60	F12 × $C$26
14	110	0.82	67.30	D13 × C14	571.31	F13 × $C$26
15	120	0.82	55.20	D14 × C15	669.39	F14 × $C$26
16	125		50.00	D15 × (0.82^0.5^)		
17	130	0.82	45.29	D16 × (C17^0.5^)	784.31	F15 × $C$26
18	140	0.82	37.15	D17 × C18	918.96	F17 × $C$26
19	150	2	74.30	D18 × C19	1076.72	F18 × $C$26
20	160	2	148.60	D19 × C20	1261.56	F19 × $C$26
21	170	2	297.20	D20 × C21	1478.14	F20 × $C$26
22	180	2	594.40	D21 × C22	1731.90	F21 × $C$26
23	190	2	1188.80	D22 × C23	2029.22	F22 × $C$26
24	200	2	2377.59	D23 × C24	2377.59	F23 × $C$26
25	GM	Product (C3:C24)^(1/20)^				
26	GM	1.17				

**Table 9:** Estimation of geometric means (based upon Ln-transformation of individual observations).

Data Row	*C*_max_ Column D	Ln *C*_max_ Column E	Ln *C*_max_
Row 2	11	2.40	-
Row 3	7	1.95	
Row 4	9	2.20	
Row 5	4	1.39	
Row 6	10	2.30	
Row 7	12	2.48	
Row 8	23	3.14	
Row 9	15	2.71	
Row 10	7	1.95	
Row 11	18	2.89	
Arithmetic mean	11.6	2.34	sum(E2:E11)/10
Geometric mean (exponentiation of arithmetic mean of Ln values))	-	10.4	exp[sum(E2:E11)/10]

**Table 10:** Values and spreadsheet code for estimating the geometric stdevs.

		Column A	Column B	Column C	Column D
Row		Number	Ln number	Geometric sq′d dev	
1	-	11	2.40	0.00	(B1–D12)
2		7	1.95	0.15	(B2–D12)^2^
3		9	2.20	0.02	(B3–D12)^2^
4		4	1.39	0.91	(B4–D12)^2^
5		10	2.30	0.00	(B5–D12)^2^
6		12	2.48	0.02	(B6–D12)^2^
7		23	3.14	0.63	(B7–D12)^2^
8		15	2.71	0.14	(B8–D12)^2^
9		7	1.95	0.15	(B9–D12)^2^
10		18	2.89	0.30	(B10–D12)^2^
11	Sum	-	-	2.34	sum(D1:D10)
12	Average			-	sum(B1:B10)/10
13	Geometric mean				exp(D12)
14	Arithmetic Stdev				-
14	Geometric Stdev			5.29	D13 × sqrt(D11/9)

**Table 11:** Arithmetic means versus LSmeans for the effect of exercise on body weights.

	**No exercise group**		**Exercise group**	
	Male	Female	Male	Female
	210	150	200	138
	215	168	192	138
	189	145	176	144
	196	160	202	154
	202	166	210	140
		155	189	
		159	176	
		149	188	
		138	192	
		188		
Marginal means	202	158	192	143
Arithmetic mean	173		174	
LSmean	180		167	

**Table 12:** Comparison of stdev based upon an assumption of a single versus bimodal dataset comprising the treatment effect.

LSmean	Description	Within/between calculated Stdev	Simulated Stdev (equal probability of sampling from each group)
No exercise (T0)	Average of T0 males and T0 females	25.70	25.10
Exercise (T1)	Average of T1 males and T1 females	27.77	27.05
Males	Average of T0 males and T1 males	12.57	12.95
Females	Average of T0 females and T1 females	13.71	13.93

**Table 13:** Example of a cross study assessment of mean and stdev. MPH: miles per hour.

Heading	Trainer	# Runners	Average MPH	Stdev	Mean × ni	LSmean
	1	10	6.2	1.24	62	-
	2	5	5.5	0.55	27.5	-
	3	8	6.1	0.915	48.8	-
	4	12	6.8	1.7	81.6	-
Sum	-	35	-	-	219.9	6.28

**Table 14:** Total weighted stdev across the four trainers.

Data Row	Trainer Col B	# Runners Col C	Average MPH Col D	Stdev Col E	Mean×ni Col F	Lsmean Col G	*SS_W_* Col H	*SS_B_* Col I	*Variance_WB_* Col J	Stdev_WB_ Col K
Row 2	1	10	6.2	1.24	62	-	13.84	0.07	-	-
Row 3	2	5	5.5	0.55	27.5		1.21	3.06		
Row 4	3	8	6.1	0.915	48.8		5.86	0.27		
Row 5	4	12	6.8	1.7	81.6		31.79	3.21		
Row 6	-	35	-	-	219.9	6.28	52.70	6.61	1.74	1.32

**Table 15:** Excel cell formulas.

Data Row	Trainer Col B	# Runners Col C	Ave MPH Col D	Stdev Col E	Mean×ni Col F	Lsmean Col G	*SS_W_* Col H	*SS_B_* Col I	*Variance_WB_* Col J	Stdev_WB_ Col K
Row 2	1	10	6.2	1.24	D2 × C2	-	(E2^2^) × (C2-1)	C2 × (D2-$G$6)^2^	-	
Row 3	2	5	5.5	0.55	D3 × C3		(E3^2^) × (C3-1)	C3 × (D3-$G$6)^2^		
Row 4	3	8	6.1	0.915	D4 × C4		(E4^2^) × (C4-1)	C4 × (D4-$G$6)^2^		
Row 5	4	12	6.8	1.7	D5 × C5		(E5^2^) × (C5-1)	C5 × (D5-$G$6)^2^		
Row 6	-	SUM (C2:C5)	-	-	SUM (F2:F5)	F6/C6	SUM (H2:H5)	SUM (I2:I5)	(I6 + H6)/(C6 − 1)	SQRT(J6)

**Figure A1:**
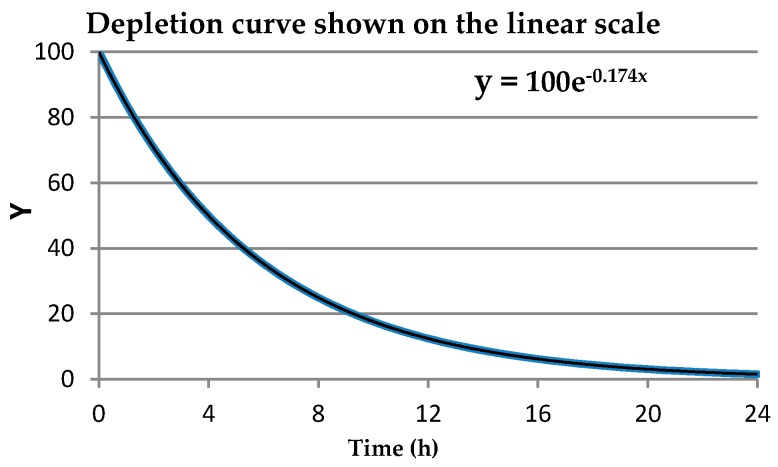
Depletion curve shown on the linear scale. The curve is best described by the relationship: *Y* = 100 × exp^−0.1735x^; where *X* = time (h).

**Figure A2:**
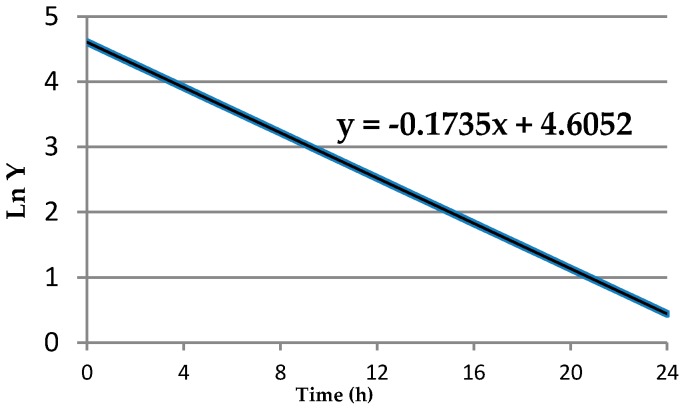
Depletion curve shown for Ln-transformed values. The line is best described by the equation Ln(*Y*) = −0.1735*X* + 4.6052, where *X* = time (h).

## Appendix A

Types of means discussed in this article (refer to references as defined in the body of the manuscript):

(A1) $$\mathrm{Arithmetic}\;\mathrm{mean}\;=\;\frac{sum}{n}$$

(A2) $$\mathrm{Geometric}\;\mathrm{mean}\;=\;\sqrt[{n}]{product}\;(e.g.,\;\mathrm{growth}\;\mathrm{rate})$$

(A3) $$\mathrm{Harmonic}\;\mathrm{mean}\;=\;\frac{n}{\left[\frac{1}{a}+\frac{1}{b}+\frac{1}{c}...\frac{1}{n}\right]}$$

Least square means are estimated in a manner comparable to that of the arithmetic mean. However, the fundamental difference is that the study design and potential imbalance (dissimilarity between the numbers of observations in the comparators) are adjusted for, such that each observation becomes equally weighted [7]. This is accomplished by the use of marginal means. For example, for two groups of size *n*_1_ and *n*_2_, with marginal means ${\bar{X}}_{1}$ and ${\bar{X}}_{2}$:

(A4) $$\mathrm{Least}\;\mathrm{squares}\;\mathrm{mean}\;=\;(n_{1}\times {\bar{X}}_{1}+n_{2}\times {\bar{X}}_{2})/(n_{1}+n_{2})$$

For more information on the challenges associated with the use of a spreadsheet to estimate the LSmeans and the lack of a simple formula to follow, please see the blog discussion link: http://onbiostatistics.blogspot.com/2009/04/least-squares-means-marginal-means-vs.html.

With two or more factors in a model, the least squares mean calculation is depicted in matrix algebra which is beyond the scope of this manuscript. For more information, refer to [10].

## Appendix B

Proof that the arithmetic mean is always greater than the geometric mean, which is always greater than the harmonic mean:

(A5) $$\mathrm{Arithmetic}\;\mathrm{mean}\;=\;\frac{A+B}{2}$$

(A6) $$\mathrm{Geometric}\;\mathrm{mean}\;=\;\sqrt{AB}$$

(A7) $$\mathrm{Harmonic}\;\mathrm{mean}\;=\;\frac{2}{\left[\frac{1}{A}+\frac{1}{B}\right]}\;\mathrm{and}\;\mathrm{if}\;\mathrm{multiplied}\;\mathrm{by}\;\frac{AB}{AB}\;\mathrm{can}\;\mathrm{be}\;\mathrm{written}\;\mathrm{as}\;\frac{2AB}{B+A}$$

Demonstrating that the arithmetic mean > geometric mean:

(A8) $${\left(A-B\right)}^{2}\geq 0$$

*A*^2^ − 2*AB* + *B*^2^ ≥ 0.
(A9)

By adding 4*AB* to each side of the inequality,

(A10) $$A^{2}+2AB+B^{2}\geq 4AB,\;\mathrm{which}\;\mathrm{is}\;{\left(A+B\right)}^{2}\geq 4AB$$

Taking the square root of the both sides, *A* + *B* ≥ 2$\sqrt{AB}$ and then dividing both sides by 2,

(A11) $$\frac{A+B}{2}\geq \sqrt{AB}$$

Showing that the arithmetic mean is greater than or equal to geometric mean.

Continuing, we multiply the line above by 2, returning to *A* + *B* ≥ 2$\sqrt{AB}$. Then, divide both sides by *A* + *B*,

1 ≥ $\frac{2\sqrt{AB}}{A+B}$ and next multiply both sides by $\sqrt{AB}$

(A12) $$\sqrt{AB}\geq \frac{2AB}{A+B}$$

Showing that the geometric mean is greater than or equal to the harmonic mean.

Note that these relationships hold under the condition that *A* and *B* are non-negative values.

These results can be extended to an arbitrary number of variables [11]. However, only two are included here to facilitate understanding.

## Appendix C

### Relationship of Terminal Elimination Rate Constant and the *T*_1/2_

Starting at 1 (for 100%) as the portion of the initial amount of a chemical, the decay curve decreases to nearly zero. Using 0 to 1 on the y-axis, the half-life is the time associated with the point at which the curve crosses 0.5. Typically, the amount on the *y*-axis is transformed to its natural log (Ln) values since decay curves are curvilinear and the log transformation linearizes the relationship between the Ln fractional amount of original material remaining and time. Lambda_z (_*λ*_z_) is the elimination rate constant at the *z*^th^ phase of the decay portion of the profile, estimated as the slope of the line. For the sake of this discussion, assume that the drug in question follows a one-compartment open body model and therefore “*z*” is the terminal phase of decline.

Assume that the fractional part of the original amount at time 0 is 1, the entire original amount, which is the intercept of a (log linear) regression. Let *Y* be the fractional amount of original material remaining at time *t*, then the linear relationship is:

Ln(*Y*(*t*)) = Ln(1) − *λ*_z_ × (*t*),
(A13)

Note that since Ln(1) = 0, the regression equation is really:

Ln(*Y*(*t*)) = −*λ*_z_ × (*t*),
(A14)

The regression equation associated with the half-life would be that of the time when the value of *Y*(*t*) would be ½ or 0.5. That is,

Ln(1/2)= −*λ*_z_ × (*T*_1/2_).
(A15)

Since Ln(½) = Ln(1) − Ln(2), then upon dividing both sides by −*λ*_z_, we can solve for *T*_1/2_:

((Ln(1) − Ln(2)) /(−*λ*_z_) = *T*_1/2_ (A16)

Since Ln(1) = 0, the equation can be rewritten as:

−Ln(2)/−*λ*_z_ = *T*_1/2_ = 0.693/*λ*_z_ (A17)

The relationship between *Y*(*t*) vs. *t* and Ln(*Y*(*t*)) vs. *t* is illustrated in Figure A1 and Figure A2 and in Table A1.

**Table A1:** Comparison of arithmetic vs. HM for *T*_1/2_ estimates (including both the values and the codes for estimating these values based upon the columns and rows that might be found in an Excel spreadsheet).

Data Row	Column C	Column D	Column E			
	*T*_1/2_	*λ*_z_	1/*T*_1/2_	*T*_1/2_	0.693/*λ*_z_	*T*_1/2_
Row 2	11	0.063	0.091			
Row 3	7	0.099	0.143			
Row 4	9	0.077	0.111			
Row 5	4	0.173	0.25			
Row 6	10	0.069	0.1			
Row 7	12	0.058	0.083			
Row 8	23	0.03	0.043			
Row 9	15	0.046	0.067			
Row 10	7	0.099	0.143			
Row 11	18	0.039	0.056			
Arithmetic mean	11.6	0.075		sum(C2:C11)/10		
0.693/(Arith mean *λ*_z_)		9.202			0.693/(sum(D2:D11)/10)	
Harmonic mean of *T*_1/2_			9.202			1/(sum(E2:E11)/10)

## Appendix D

### Cross-Study Variability Estimation Procedure (Background)

It is demonstrated through the following algebra that a total variance estimate calculated by adding the squared weighted within- and between-study stdevs is biased and gives incorrect weights to the within and between-study components of variance.

In the general setting where *X_ij_* is the *j*th observation from group I, basic textbook introductions [1] to analysis of variance show that the *SS_T_*, the total sum of squared deviations from the grand mean, ${\bar{X}}_{..}$, can be divided into two sums of squares, as illustrated below, because their cross products sum to zero.

(A18) $$SS_{T}=\sum_{i=1}^{I}\sum_{j=1}^{n_{i}}\left(X_{ij}-{\bar{X}}_{..}\right){\;}^{2}=\;\sum_{i=1}^{I}\sum_{j=1}^{n_{i}}\left[\left(X_{ij}-{\bar{X}}_{i.}\right){\;}^{2}+\left({\bar{X}}_{i.}-{\bar{X}}_{..}\right){\;}^{2}\right]\;$$

The first component of the right hand side of this equation is the *SS_W_*. As discussed prior to Equation (21), by definition of the sample Stdev for study *i*, the contribution of study *i* to *SS_W_* is equal to (*n_i_* − 1) $S_{i}^{2}$ and the *SS_W_* is the sum of the contributions from all studies.

(A19) $$SS_{W}=\;\sum_{i=1}^{I}\sum_{j=1}^{n_{i}}{\left(X_{ij}-{\bar{X}}_{i.}\right)}^{2}=\sum_{i=1}^{I}\left(n_{i}-1\right)\;S_{i}^{2}$$

The second component of the right hand side of the equation describing *SS_T_* is the *SS_B_*. It can be simplified as displayed in Equation (22) because there are no terms involving the index *j*.

(A20) $$SS_{B}\;=\sum_{i=1}^{I}\sum_{j=1}^{n_{i}}{\left({\bar{X}}_{i.}-{\bar{X}}_{..}\right)}^{2}=\sum_{i=1}^{I}n_{i}\left({\bar{X}}_{i.}-{\bar{X}}_{..}\right){\;}^{2}$$

We examine the case where all the studies have the same sample size, *n*, to facilitate further discussion. (*n* × *I* = *N* total observations). When conducting a meta-analysis, we assume that all studies have drawn samples from the same population. Therefore, the average of the stdevs across the studies (${\bar{S}}_{.}$) is an estimate of stdev for the population and squaring ${\bar{S}}_{.}$ provides an estimate of the within group variance, which is the same for all groups. Under these two assumptions, (*n* − 1)${{\bar{S}}_{.}}^{2}$ can be substituted for the sum of the squared deviations for each group. Carrying this out, one finds that *SS_W_* is estimated by (*N* − *I*)${{\bar{S}}_{.}}^{2}$

(A21) $${SS}_{W}\;=\sum_{i=1}^{I}(n-1)\;{\bar{S}}_{.}^{2}\;=I(n-1){\bar{S}}_{.}^{2}\;=(N-I){\bar{S}}_{.}^{2}$$

Next, consider the between group sums of squares *SS_B_* with equal *n*.

(A22) $$SS_{B}\;=n\;\sum_{i=1}^{I}\left({\bar{X}}_{i}-{\bar{X}}_{..}\right){\;}^{2}\;$$

The definition of the sample variance among the group averages $S_{{\bar{X}}_{i}}^{2}$ is as follows.

(A23) $$S_{{\bar{X}}_{i}}^{2}\;=\;\frac{\sum_{i=1}^{I}{\left({\bar{X}}_{i}-{\bar{X}}_{..}\right)}^{2}\;}{I-1}$$

Using the same argument as the one used prior to Equation (21), the sum of squared deviations of the study means about their grand mean can be rewritten as (*I* − 1)$S_{{\bar{X}}_{i}}^{2}$.

(A24) $$SS_{B}\;=\;n\sum_{i=1}^{I}{\left({\bar{X}}_{i}-{\bar{X}}_{..}\right)}^{2}\;=\;n\;\left(I-1\right)\;S_{{\bar{X}}_{i}}^{2}\;=\;\left(N-n\right)S_{{\bar{X}}_{i}}^{2}$$

When the *SS_W_* and *SS_B_* are put back over *N* − 1 to estimate the total variance, the weights given to each component are seen more clearly.

(A25) $$\frac{{SS}_{T}}{N-1}\;=\;\frac{\left(N\;-\;I\right)}{N-1}{\bar{S}}_{.}^{2}\;+\;\frac{\left(N-n\right)}{N\;-1}S_{{\bar{X}}_{i}}^{2}$$

This shows that the contribution of the two variance components to the total variance is not equal and depends on the number of studies and the size of the studies. For a fixed total size of *N*, as the number of equal size studies increase (implying smaller *n* per study), less weight is placed on the within component and similarly, as the size of the studies increase (implying fewer studies, which results in smaller values of *i*), less weight is placed on the between component.

While it is not possible to obtain a neat form for the weights when the sample sizes vary per study, as is typically the case, the same principles apply. The equations used in the development of the unweighted method of estimating the LSmean and the total variance use the observed sample sizes and therefore use the appropriate weights for the within and between components in calculating the total population variance.
